# On Modern Progress in Ophthalmic Medicine and Surgery

**Published:** 1895-11-09

**Authors:** Robert Brudenell Carter

**Affiliations:** Consulting Ophthalmic Surgeon to St. George's Hospital.


### On Modern Progress in Ophthalmic Medicine and Surgery.

By Robert Brudenell Carter, F.R.C.S., Consulting Ophthalmic Surgeon to St. George's Hospital.

### SQUINT.

(*Continued from page 42.*)

If we study the development of ordinary convergent squint, by the light of the considerations laid down in the preceding paper, we shall usually find that the subject of it is a child whose eyes are flat in a moderate degree, and in whom the natural association between accommodation and convergence is maintained. Such a child, at the age of two or three years, begins to scrutinise carefully the appearance of near objects, of toys, pictures, and the like, and desires to obtain clear visual images of them. In order to obtain such images, he is compelled to accommodate, not only for the actual distance of the object of vision, but in the somewhat greater degree which is required to correct the flatness of his eyes; and in doing this he renders his eyes convergent to a point somewhat nearer than the position of the object, which, if he held it in front of his nose, midway between his eyes, would appear double. He soon finds, however, that this disturbing double vision is removed by holding the object a little to the right or left—that is, in front of one eye or the other, instead of between them. If we remember that the eyes are combined in a position of over convergence, we shall see that the practical effect of holding the object directly in front of the right eye will be to turn both eyes towards the right. An amount of movement in this direction which brings the convergent right eye into the middle of the eyelid opening will bring the convergent left eye well in towards the nose; and the effect will be to cast the image of the left eye upon a peripheral and comparatively insensitive part of the retina, where it may easily pass unnoticed by the consciousness, which will be engrossed by the correctly-placed image received by the right eye. If the object, instead of being held in front of the right eye, is held in front of the left, a corresponding effect will be produced, with the difference that the left eye will appear to be properly directed, and the right eye to squint. The left eye will then receive the central image, which must be regarded, and the right will receive the peripheral image which may be neglected. The eyes in either case may be distinguished as the working eye and the squinting eye; but it must not be forgotten that both, and not merely the latter, are misdirected. The working eye is an eye tending towards convergence, and forcibly turned towards its temporal side while its convergence muscle remains in action.

In these circumstances parents, if moderately observant (a condition not invariably fulfilled), will be likely to notice that the child squints when he looks attentively at any near object. When he ceases to do this his eyes return towards the correct position, and for a time the squint might be described as only occasional. The conditions which gave rise to it remaining, and the attention being more and more directed to the details of his environment, the squint becomes more and more permanent and more and more pronounced. If the eyes are equal in degree of flatness, in acuteness of vision, and in muscular power, it will be a matter almost of accident whether one or other of them is employed on any given occasion, and, in such circumstances, the squint is said to be "alternating." In most cases these conditions are not fulfilled, and, for some reason or other, it is more easy to employ one eye than its fellow. The eye most easily used will then be used habitually, and will become the working, while the other will become the squinting eye. The squint is no longer alternating, but "fixed."

A flat, or, in other words, a small eye, may perhaps be taken to represent a comparatively undeveloped organ, and it is certain not only that the vision of such eyes is often below the normal standard of acuteness, but that the vision of one of them is often less acute than that of its fellow. When this is so, the better sighted becomes also the working eye, and the vision of the squinting eye often suffers further deterioration, presumably from the almost complete withdrawal of mental attention from the images which it receives. It is not uncommon, even at an early age, to find the squinting eye practically blind, although it would appear that it must originally have been sighted in order that the squint should be produced. An eye which loses vision from disease is apt to become divergent from want of visual guidance, but I have never seen it become convergent.

In considering what should be done with a young child who squints, the question of the equality or disparity of vision in the two eyes becomes one of primary importance. Speaking generally, when vision is equal in the two, it is better to defer operation until at least the age of eight years. Perfection of the operative result can only be attained, subsequently to the comparatively rough correction effected by tenotomy, by the unconscious efforts of the patient to combine the eyes for the avoidance of double vision, and such efforts are hardly made until the faculty of attention and the muscular power are well developed. But, if the habitually disused eye possess only defective vision, it must be borne in mind that this vision may improve if the eye be brought into use, and that it may undergo further deterioration if the eye remains disused. Neither condition is certain, neither can be rejected as impossible. In such instances, therefore, it is better to operate at however early an age, even if the attainment of perfect position should require a second operation at a later period. The vision of a young child cannot be accurately tested by anyone who is not quite familiar with him. I advise parents to do it by tying a small patch over one eye as a bit of play, perhaps doing it upon themselves, or upon a doll, until the imitativeness of childhood is aroused. When the child can be induced, by any nursery contrivance, to submit to have one eye tied up, it will be quite easy to observe whether he will see and find any small object, such as a toy or a sweetmeat, with equal readiness with either. Should he do so, the operation should always be deferred. Should he fail to do so, with such constancy as to establish the existence of defective sight in the squinting eye, this should always have the benefit of such a chance of improvement as the early performance of the operation may afford. I have occasionally seen almost complete restoration of vision; but, as a general rule, there is not very much to be gained. Even in these cases we may hope that further impairment has been arrested.

